# Role of Polysomnography in Tracheostomy Decannulation in Neuromuscular Disease: A Case Report

**DOI:** 10.7759/cureus.99829

**Published:** 2025-12-22

**Authors:** Patrícia Pereira, Ana Luísa Vieira, Sónia Tizón

**Affiliations:** 1 Physical Medicine and Rehabilitation, Hospital de Braga, Braga, PRT; 2 Pulmonology, Hospital de Braga, Braga, PRT

**Keywords:** hypoventilation, neuromuscular disease, non-invasive ventilation, polysomnography, tracheostomy decannulation

## Abstract

Tracheostomy decannulation in patients with neuromuscular disease presents significant challenges due to impaired airway clearance, pharyngolaryngeal hypotonia, and chronic hypoventilation. Polysomnography (PSG) may provide an objective assessment of ventilatory stability and support safe decision-making. We describe a 54-year-old woman with sequelae of Arnold-Chiari malformation type I, syringomyelia, and tetraplegia (AIS D, neurological level C1), who developed chronic respiratory insufficiency requiring tracheostomy. Despite achieving nocturnal normocapnia with non-invasive ventilation, she exhibited persistent daytime hypercapnia and severely impaired cough. A capped tracheostomy PSG performed under nasal-mask non-invasive ventilation demonstrated no worsening of baseline hypoventilation, confirming ventilatory stability and supporting decannulation readiness. Anxiety delayed the procedure, but multidisciplinary evaluation continued to indicate safety for future decannulation. This case highlights the utility of PSG in the assessment of complex neuromuscular patients with multifactorial hypoventilation, where standard pulmonary function testing may be limited.

## Introduction

Neuromuscular diseases frequently impair respiratory muscle strength, reduce lung volumes, and compromise airway clearance. These abnormalities predispose patients to sleep-disordered breathing and chronic hypoventilation, often necessitating tracheostomy for ventilatory support and airway protection [1,2]. Determining readiness for tracheostomy decannulation in this population is complex, particularly when daytime clinical assessment and arterial blood gases fail to predict nocturnal ventilatory stability.

Polysomnography (PSG) provides a comprehensive nocturnal assessment of airflow, respiratory effort, oxygen saturation, and carbon dioxide trends. In adults with neuromuscular disease, the purpose of PSG prior to decannulation is not necessarily to eliminate obstructive events, but to confirm that ventilatory support, most commonly non-invasive ventilation (NIV), can maintain adequate gas exchange during sleep under conditions simulating post-decannulation physiology [3,4]. This differs from pediatric decannulation paradigms, in which PSG is often used to identify surgically correctable airway obstruction. Capped-tracheostomy PSG may therefore be particularly valuable when pulmonary function testing is not feasible, or airway anatomy is compromised [5].

We present a case illustrating how PSG informed decannulation decision-making in a patient with complex neuromuscular respiratory insufficiency following Arnold-Chiari malformation type I.

## Case presentation

A 54-year-old woman with a history of Arnold-Chiari malformation type I and syringomyelia experienced abrupt neurological deterioration in September 2020. She underwent emergent suboccipital decompressive craniotomy and C1 laminectomy. Postoperatively, she developed tetraplegia (American Spinal Injury Association Impairment Scale (AIS) D, neurological level C1) and multifactorial hypoventilation attributed to impaired central respiratory drive, neuromuscular weakness, and deconditioning.

From September 17, 2020, to March 15, 2021, she required prolonged hospitalization for management of respiratory insufficiency. She was discharged to inpatient rehabilitation with nocturnal bilevel positive airway pressure ventilation (inspiratory positive airway pressure 22 cmH₂O, expiratory positive airway pressure 6 cmH₂O, respiratory rate 16 breaths/min, inspiratory time 1.5 s) and mechanical insufflation-exsufflation (+40/−40 cmH₂O). Her initial tracheostomy was closed prior to discharge.

On March 17, 2021, she was readmitted with acute-on-chronic hypercapnic respiratory failure. After optimization of ventilatory support, she was again discharged on nocturnal NIV. On April 16, 2021, she developed recurrent respiratory failure associated with upper-airway obstruction caused by marked pharyngolaryngeal hypotonia, bilateral vocal cord hypomotility, and epiglottic collapse. A tracheostomy and percutaneous endoscopic gastrostomy were performed on April 28, 2021.

Nasofibrolaryngoscopy (NFL) on May 6, 2021, revealed bilateral vocal cord paralysis in the median position, absent cough reflex, significant salivary stasis, and visible aspiration. Repeat evaluation on May 28, 2021, demonstrated moderate dysphagia (Macedo and Filho grade II) with reduced laryngeal sensitivity but no frank aspiration. The Functional Oral Intake Scale (FOIS) assessment confirmed the need for supplemental enteral feeding.

**Figure 1:**
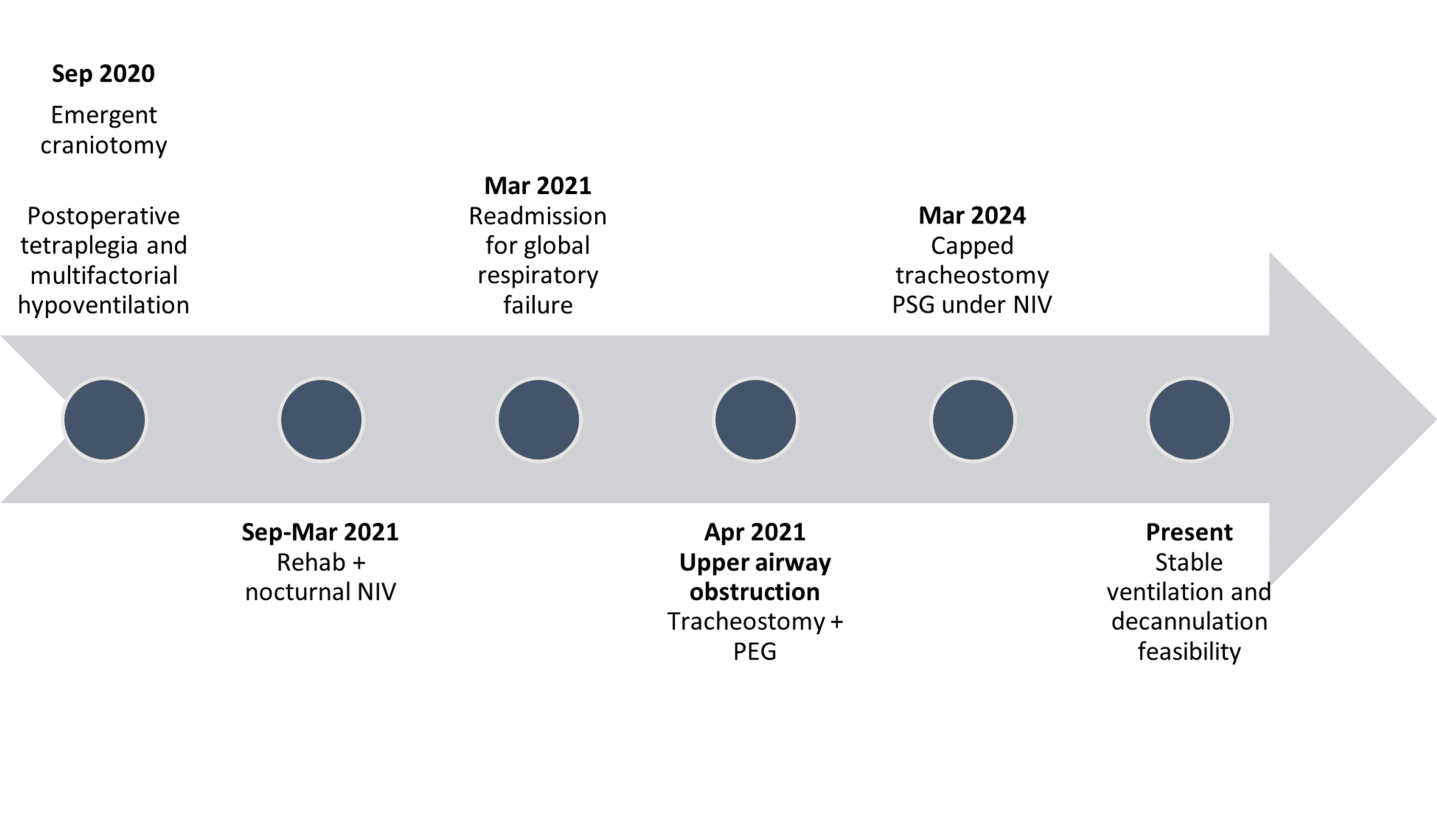
Case presentation timeline Rehab: rehabilitation; NIV: non-invasive ventilation; PEG: percutaneous endoscopic gastrostomy; PSG: polysomnography Figure created by the authors with Microsoft PowerPoint (Microsoft Corp., USA)

Recurrent bleeding during cannula changes was attributed to a tracheal lesion near the cuff and treated with cauterization. The tracheostomy was replaced with a fenestrated, cuffed size 6 cannula. Despite achieving nocturnal normocapnia on NIV, the patient exhibited persistent daytime hypercapnia with transcutaneous carbon dioxide (TcCO₂) values around 65 mmHg. Peak cough flow was 0 L/min, indicating severely impaired airway clearance. Pulmonary function testing was not feasible due to interface limitations (Figure 1).

**Figure 2:**
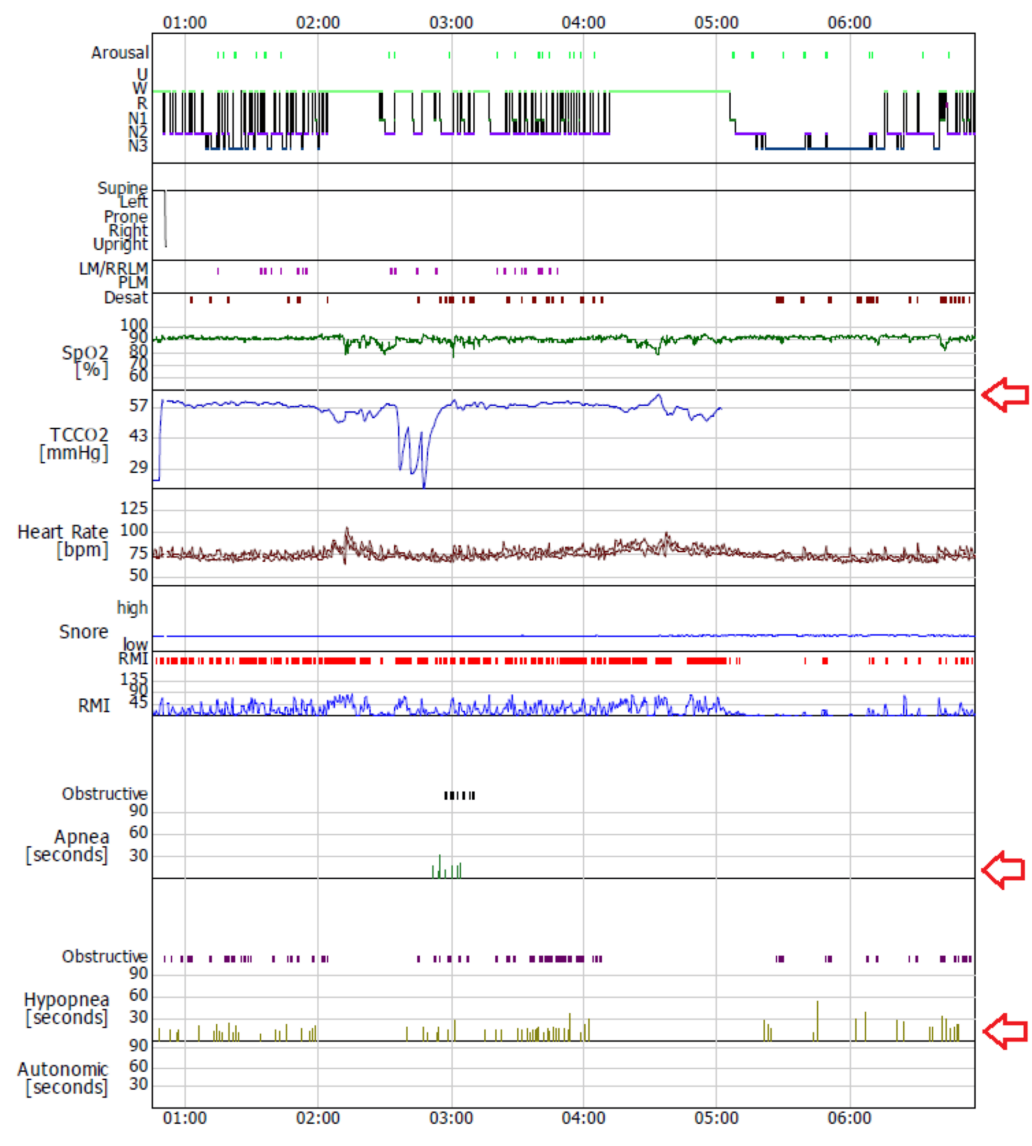
Polysomnography (PSG) summary graph The arrows indicate no worsening of baseline hypoventilation and stable transcutaneous CO₂.

Given consideration for decannulation, a capped-tracheostomy PSG was performed to assess nocturnal ventilatory safety (Figure 2, Table 1). The tracheostomy cannula was capped with the cuff completely deflated, allowing airflow through the upper airway and simulating post-decannulation physiology. Non-invasive ventilation was delivered via a nasal mask throughout the study.

**Table 1:** Polysomnography (PSG) summary table

Parameter	Value	Reference values
Apnea + Hypopnea Index	21.6/h	Normal <5 events/hour
Apnea Index	1.9/h	Normal: < 5 events/hour
Hypopnea Index	19.7/h	Normal: < 5 events/hour
Oxygen Desaturation Events	11.2/h	Normal: < 5-10 events/hour
Mean SpO₂	91.4%	Normal mean sleeping SpO₂: ≥ 94% Mild reductions: 92–94%
Saturation <90%	25.9 min (11.9%)	Normal < 10%
Arousal Index	7.4/h	Normal < 5–10 arousals/hour

PSG demonstrated consolidated sleep with 4.8% stage N1, 35.6% stage N2, 19.5% stage N3, and 0.1% rapid eye movement sleep. The Apnea-Hypopnea Index (AHI) was 21.6 events/h, predominantly obstructive hypopneas (19.7 events/h). Mean oxygen saturation was 91.4%, with a nadir of 77%, an oxygen desaturation index of 11.2 events/h, and time with saturation below 90% corresponding to 11.9% of total sleep time. Importantly, TcCO₂ remained stable throughout the night, with no worsening of baseline hypoventilation.

Although moderate obstructive events were observed, they were not associated with progressive hypercapnia or sustained oxygen desaturation. Given that the capped tracheostomy itself imposes additional airway resistance, the ability of NIV to maintain stable gas exchange under these conditions suggested that ventilatory performance after decannulation would likely be equal or superior.

Based on these findings, the multidisciplinary team (pulmonology, otolaryngology, and rehabilitation medicine) concluded that decannulation was physiologically feasible. The procedure was temporarily deferred due to patient anxiety rather than medical contraindication. Ongoing follow-up confirmed clinical stability, effective NIV use, tolerance of a speaking valve, and preserved oral intake. She remains under periodic reassessment for future decannulation.

## Discussion

Assessing readiness for tracheostomy decannulation in patients with neuromuscular disease is particularly challenging. Weak cough, impaired airway clearance, and sleep-related hypoventilation all contribute to uncertainty in predicting post-decannulation respiratory stability. Daytime blood gases, clinical examination, and endoscopic airway evaluation may not reflect nocturnal respiratory physiology [1,2].

PSG provides an objective assessment of ventilation, oxygenation, and carbon dioxide control during sleep. In contrast to pediatric decannulation protocols, where PSG is often used to identify surgically correctable upper-airway obstruction, adult neuromuscular patients frequently exhibit functional airway compromise that is not amenable to operative intervention [3,6-10]. In these patients, the goal of PSG is not to normalize the AHI, but to determine whether NIV can adequately compensate for respiratory muscle weakness and upper-airway hypotonia.

In this case, PSG performed with the tracheostomy capped and cuff deflated demonstrated stable nocturnal ventilation under NIV, despite the presence of moderate obstructive hypopneas. The absence of nocturnal hypercapnia was the critical finding supporting decannulation readiness. Importantly, the capped tracheostomy represents a physiologically disadvantageous condition due to added airway resistance; therefore, effective ventilation under these circumstances provides additional reassurance regarding post-decannulation safety.

This case underscores the utility of PSG when pulmonary function testing is not feasible and highlights its role in multidisciplinary decision-making. Even when decannulation is delayed by non-medical factors, PSG can distinguish physiological readiness from psychosocial barriers and support clinician confidence in NIV-based decannulation strategies.

## Conclusions

PSG can play a decisive role in assessing tracheostomy decannulation readiness in adults with neuromuscular disease and complex respiratory insufficiency. In patients for whom conventional clinical assessments and pulmonary function testing are limited or unreliable, PSG provides an objective evaluation of nocturnal ventilation, airway stability, and gas exchange under conditions that closely approximate the post-decannulation state.

By confirming ventilatory adequacy and carbon dioxide control while the tracheostomy is occluded, PSG complements endoscopic findings and multidisciplinary clinical judgment. Incorporating PSG into the decannulation assessment process may enhance patient safety and support individualized, evidence-based decision-making in high-risk neuromuscular populations.
